# Psychedelics as Novel Therapeutics for Chronic Pain in Veterinary Medicine: A Hypothesis-Driven Protocol Using Low-Dose 1-Cyclopropionyl-D-lysergic Acid Diethylamide (1cp-LSD) in Canine Osteoarthritis

**DOI:** 10.3390/ani16010003

**Published:** 2025-12-19

**Authors:** Elisa Hernández-Álvarez, Andrea Acosta-Dacal, Octavio P. Luzardo, Luis Alberto Henríquez-Hernández

**Affiliations:** 1 Unit of Toxicology, Clinical Sciences Department, Universidad de Las Palmas de Gran Canaria, 35016 Las Palmas de Gran Canaria, Spain; andrea.acosta@ulpgc.es (A.A.-D.); octavio.perez@ulpgc.es (O.P.L.); luis.henriquez@ulpgc.es (L.A.H.-H.); 2 Universidad Fernando Pessoa Canarias, 35450 Guía, Spain; 3 Asociación Científica Psicodélica, 35412 Arucas, Spain

**Keywords:** pain management, chronic pain, veterinary therapeutics, osteoarthritis, psychedelics, 1cp-LSD, analgesic, caregiver expectations

## Abstract

Low-dose psychedelics have shown potential in modulating chronic pain in humans, yet their application in veterinary medicine remains unexplored. This study protocol proposes to investigate the therapeutic potential of low-dose oral administration of 1-cyclopropionyl-D-lysergic acid diethylamide (1cp-LSD), a legal LSD analogue in certain countries, for the management of chronic pain in privately owned dogs with osteoarthritis. The study will employ a randomized, placebo-controlled design with caregivers blinded to treatment allocation. A cohort of about 24 dogs previously diagnosed with osteoarthritis, will orally receive sub-perceptual, intermittent doses of 1cp-LSD over a 30-day period, while maintaining their standard analgesic regimens to safeguard animal welfare. Outcome measures will include the Canine Brief Pain Inventory and caregiver-reported assessments, including the Treatment Expectation Questionnaire (TEX-Q), to evaluate both pharmacological efficacy and the influence of caregiver expectations as an indirect indicator of placebo effects as a secondary aim. The study anticipates a reduction in pain scores among treated dogs, potentially modulated by caregiver expectations. However, the sustained effect of 1cp-LSD in osteoarthritis remains uncertain due to interactions with inflammatory mediators. Limitations include the lack of established dose–response relationships, small cohort size, and variability in caregiver perceptions, which will be analyzed descriptively. The protocol establishes a comprehensive and methodologically framework to evaluate both the pharmacological therapeutic effects of low-dose psychedelics in managing chronic osteoarthritic pain and the psychological factors that may influence perceived outcomes.

## Simple Summary

Osteoarthritis is a common condition in dogs that causes chronic pain and reduces quality of life. Current treatments often provide incomplete relief, highlighting the need for novel approaches. This study protocol proposes to explore the potential of psychedelics as an adjunctive strategy for managing pain in dogs with osteoarthritis. Dogs would receive small, intermittent doses of 1-cyclopropionyl-D-lysergic acid diethylamide (1cp-LSD), a legal LSD analog in certain countries, over a one-month period, while continuing their standard pain management treatments. Caregivers would report on their pets’ pain and well-being using structured questionnaires, which also capture expectations regarding the treatment. This study protocol aims to evaluate whether low-dose 1cp-LSD could reduce pain, and to understand how caregivers’ expectations might influence their perception of their pets’ condition. The planned pilot trial is expected to provide preliminary insights into the safety and potential benefits of this approach, while identifying factors that could affect caregiver-reported outcomes. Ultimately, this protocol could help guide future research into innovative treatments for chronic pain in companion animals, contributing to better veterinary care and animal welfare.

## 1. Background

Osteoarthritis (OA) is a non-infectious, degenerative joint disease characterized by progressive hyaline cartilage degradation, osteophyte formation, and fibrosis of periarticular tissues [1]. In dogs, OA is predominantly secondary to structural instabilities such as cranial cruciate ligament rupture, malalignment following fractures, abnormal weight distribution (as seen in hip or elbow dysplasia), or previous joint disorders including septic arthritis [2,3]. OA represents the most prevalent joint pathology in veterinary medicine [4], with an estimated radiographic prevalence of 38–40% in the canine population [5,6]. Chronic pain is the most significant clinical limitation of canine osteoarthritis, as it progressively diminishes mobility, disrupts normal behavior, and negatively impacts the animal’s overall quality of life [7]. Pain can hinder routine activities such as walking, playing, or even basic elimination behaviors, and often leads to changes in the dog’s temperament, further compromising its welfare. Simultaneously, owners frequently experience emotional distress, including the perception of their pet’s suffering, as well as disruptions to their daily lives due to the increased care requirements of their affected animals [7,8]. Diagnosis of OA relies on clinical and orthopedic evaluation, supported by complementary radiographic assessment to determine disease severity [9,10]. Grading the disease is essential for monitoring its progression and guiding therapeutic strategies, particularly in the management of chronic pain. Based on radiographic findings, OA can be classified into four grades of severity [10,11]: Grade 0 (Absent): No radiographic signs of OA; Grade 1 (Mild): Minimal osteophyte formation at joint margins; no significant joint space alterations; Grade 2 (Moderate): Evident osteophytes, subchondral sclerosis, and mild joint space reduction; or Grade 3 (Severe): Marked loss of joint space, prominent osteophytes, advanced sclerosis, and possible bone deformation.

### 1.1. Pharmacological Treatment

OA is a chronic and painful condition involving nociceptive, inflammatory, and neuropathic mechanisms [12]. Briefly, persistent joint inflammation leads to peripheral sensitization of nociceptors, while long-standing stimulation induces central sensitization within the spinal cord and brain [12]. These neuroplastic changes—characterized by altered pain thresholds, spontaneous neuronal firing, and impaired inhibitory pathways—contribute to neuropathic pain components commonly observed in advanced OA. The disproportionate and persistent pain experienced by dogs with osteoarthritis can be partly explained by mechanisms of peripheral and central sensitization [13]. Peripheral sensitization involves increased excitability of nociceptive neurons due to inflammatory mediators such as bradykinin, prostaglandins, and cytokines in the joint environment, which lower the activation threshold of pain fibers [14]. Central sensitization refers to enhanced responsiveness of neurons within the central nervous system, leading to amplification of pain signals [14]. Together, these processes result in clinical signs of hyperalgesia (exaggerated pain response to noxious stimuli) and allodynia (pain elicited by normally non-painful stimuli) [12,13]. Effective management requires a multimodal analgesic approach, targeting different pain pathways when monotherapy is insufficient [15]. Current pharmacological options for canine osteoarthritis include various drug classes differing in mechanisms of action, effectiveness, and safety profiles [16,17] (Table 1). NSAIDs remain the first-line treatment, exerting analgesic effects through cyclooxygenase (COX) inhibition. Common veterinary NSAIDs include meloxicam, carprofen, and firocoxib, while COX-2–selective agents (robenacoxib, mavacoxib, cimicoxib, enflicoxib) reduce some gastrointestinal risks but all may still cause adverse events such as vomiting, diarrhea, or, rarely, renal toxicity [18]. EP4 receptor antagonists (e.g., grapiprant) offer targeted prostaglandin E2 blockade with generally mild gastrointestinal effects (Table 1).

Monoclonal antibodies targeting nerve growth factor (NGF), such as bedinvetmab, address a key mediator of nociceptor sensitization and show promising early results, though access and cost remain limiting factors [19]. Opioids provide variable analgesic efficacy, with tramadol being particularly notable due to inconsistent metabolism to its active metabolite O-desmethyltramadol (M1). In fact, the use of tramadol alone has not demonstrated clinically meaningful analgesic effects in dogs with osteoarthritis [20,21]. Moreover, unlike some other opioids, tramadol also acts as a serotonin and noradrenaline reuptake inhibitor [22], which may influence its analgesic profile and interaction potential. Due to concerns about tolerance, dependence, and side effects, opioids are generally reserved for acute pain scenarios [23]. Corticosteroids offer potent anti-inflammatory effects but are controversial for long-term OA management because of risks of cartilage damage and systemic adverse events. Emerging options include gabapentinoids, NMDA antagonists (amantadine, memantine), cannabinoids, tricyclic antidepressants, and gene-therapy approaches, though evidence for their long-term efficacy in canine chronic pain remains limited (Table 1) [16].

Despite this therapeutic diversity, current pharmacological strategies often fail because they do not address the underlying mechanisms of chronicity in osteoarthritis, such as peripheral and central sensitization, neuroplastic changes, and the affective dimension of pain [24,25,26]. Existing therapies primarily provide symptomatic relief without reversing joint damage [27], are mainly symptomatic [16], and are limited by side effects, inconsistent efficacy, and practical challenges [18,28,29]. This gap between the pathophysiology of chronic pain and available treatments underscores the unmet need for novel approaches capable of targeting both nociceptive and affective components of chronic pain—an area in which serotonergic psychedelics may hold promise as emerging neuromodulatory agents.

**Table 1:** Main pharmacological treatments currently used for pain management in canine osteoarthritis.

Drug Class	Mechanism of Action	Common Examples	Main Adverse Effects	Limitations/Remarks
NSAIDs ^1^	Inhibition of COX-1/COX-2 and reduction in prostaglandin synthesis	Meloxicam, carprofen, firocoxib	Gastrointestinal upset, renal toxicity	First-line therapy; efficacy proven but chronic use limited by adverse effects
EP4 antagonists ^2^	Blockade of prostaglandin E2 receptor EP4	Grapiprant	Vomiting, diarrhea, hyporexia (usually mild)	Effective for mild–moderate pain; limited experience in severe OA
Anti-NGF monoclonal antibodies ^3^	Neutralization of nerve growth factor to reduce nociceptor sensitization	Bedinvetmab	Rare and mild; costly	Promising novel approach but limited veterinary evidence
Opioids ^4^	μ-opioid receptor agonism	Buprenorphine	Sedation, gastrointestinal signs, tolerance	Control of acute pain
Corticosteroids ^5^	Anti-inflammatory gene modulation	Prednisolone, dexamethasone	Cartilage damage, endocrine effects	Long-term use discouraged; intra-articular use debated
Analgesic adjuvant ^6^	Various (neuromodulation, cannabinoid, NMDA antagonism, inhibition of serotonin and noradrenaline reuptake, etc.)	Gabapentin, pregabalin, amantadine, CBD, amitriptyline, tramadol	Limited data available	Promising but experimental in dogs

Abbreviations: CBD, cannabidiol; COX, cyclooxygenase; NGF, nerve growth factor; NMDA, N-methyl-D-aspartate; NSAIDs, non-steroidal anti-inflammatory drugs; OA, osteoarthritis. Supporting references: ^1^ [30,31]; ^2^ [32]; ^3^ [33]; ^4^ [23]; ^5^ [34,35]; ^6^ [15,19,36,37].

### 1.2. The Anti-Inflammatory Dimension of Psychedelics

Psychedelic compounds comprise a broad group of molecules best known for their profound psychoactive properties [38]. Initially developed for psychiatric purposes and widely explored during the 1950s, their clinical use was abruptly halted after prohibition in the 1960s, despite their relatively low toxicity and promising therapeutic potential [39]. Psychedelics can be classified according to their origin as natural (e.g., mescaline, psilocybin, dimethyltryptamine) or synthetic (e.g., MDMA, phencyclidine, ketamine); or by their pharmacological profile: classical psychedelics, which primarily act as serotonin 5-HT_2_A receptor agonists (e.g., LSD, psilocybin, DMT, mescaline), versus non-classical compounds with broader or mixed mechanisms (e.g., salvinorin A, ketamine, MDMA) [40].

**Figure 1:**
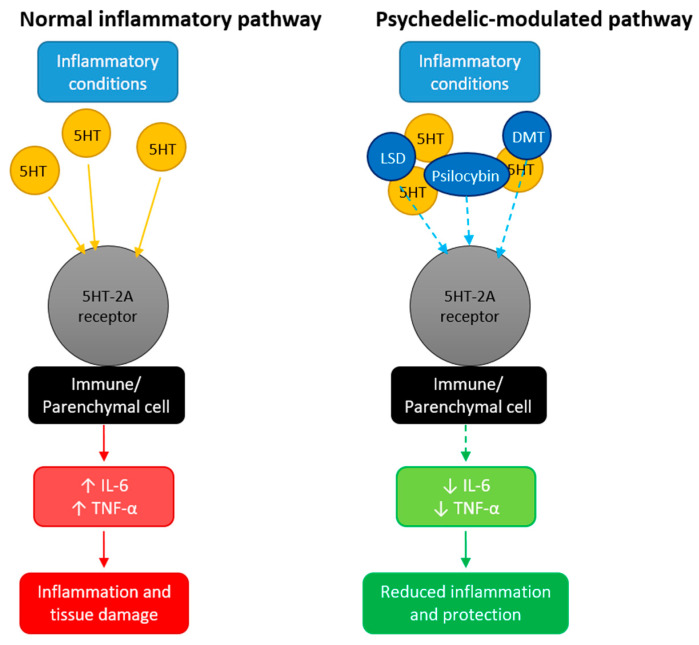
Hypothesized anti-inflammatory action of classic psychedelics through the serotonin 5-HT_2_A receptor. The left panel shows normal inflammatory conditions with elevated serotonin (5HT) levels leading to increased IL-6 and TNF-α production. The right panel shows how classical psychedelics modulate 5-HT_2_A receptor signaling at sub-psychoactive doses, selectively reducing proinflammatory cytokine expression without broad immunosupression. Solid arrows indicate normal signaling; dashed lines represent inhibitory modulation by psychedelics; white arrows indicate an increase (↑) or decrease (↓) in the levels of inflammatory mediators.

Although current research focuses mainly on their psychiatric applications [41,42], recent studies suggest that psychedelics may also exert anti-inflammatory effects (Figure 1). The 5-HT_2_A receptor, widely expressed across multiple cell types and tissues, plays a key role in the modulation of inflammation [43]. Under inflammatory conditions, serotonin levels rise, promoting the release of proinflammatory cytokines such as interleukin-6 (IL-6) and tumor necrosis factor-α (TNF-α) [44]. Pharmacological antagonism of 5-HT_2_A can inhibit these responses [45]. Interestingly, despite acting through 5-HT_2_A activation, classical psychedelics appear to attenuate inflammatory signaling, an effect observed both in vitro and in animal models at doses far below those required to elicit perceptual or behavioral changes [44,46]. These findings suggest that psychedelics modulate, rather than suppress, immune function—selectively reducing proinflammatory mediator release. As such, they may represent a novel class of oral anti-inflammatory agents with broad therapeutic potential [45,47].

### 1.3. Psychedelics as Pain Modulators

Inflammation is physiologically perceived as pain; therefore, chronic inflammation often leads to chronic pain. This condition arises from complex mechanisms involving sensitization of both peripheral and central nociceptive circuits [48]. Psychedelics acting on serotonin 5-HT_2_A receptors can modify the functional connectivity of brain regions related to pain perception, offering a potential therapeutic pathway for neuropathic states [48]. Their potential has been explored in migraine, cluster headache, fibromyalgia, phantom limb pain, and cancer-related pain [48,49,50]. Positive outcomes have also been reported in oncologic pain, suggesting that psychedelic-assisted therapy may influence both the sensory and emotional dimensions of pain [49,50].

Different mechanisms may explain the analgesic effects of psychedelics. At the molecular level, several studies show modulation of genes linked to inflammatory signaling [48]. Psychedelics promote neuroplasticity in the prefrontal cortex and amygdala, reshaping the emotional processing of pain. Their action on descending serotonergic pathways also contributes to pain modulation, together with immunomodulatory and anti-inflammatory effects [48]. Recent hypotheses, supported by functional MRI studies, suggest that psychedelics induce neuroplastic and anti-inflammatory changes that transiently disintegrate established neural networks while promoting new long-range connections that restore balance between pain and non-pain pathways [51,52]. Although the exact mechanisms remain uncertain, current evidence suggests that psychedelics provide analgesic benefits through serotonin receptor agonism combined with selective anti-inflammatory actions. Enhanced neuroplasticity and global connectivity may disrupt maladaptive pain circuits, though robust evidence beyond headache, oncologic, and neuropathic pain—such as in fibromyalgia—remains limited. In veterinary medicine, psychedelics have only been investigated for anxiety management with promising results [53,54,55], but their potential use in chronic pain in dogs remains unexplored.

### 1.4. Human Subjectivity in the Assessment of Canine Pain

While the primary aim of this proposal is to assess the therapeutic potential of low-dose 1cp-LSD in managing chronic pain in dogs with osteoarthritis, the influence of caregiver perception on pain assessment constitutes a critical secondary consideration. Behavioral changes associated with chronic pain often develop gradually and are subtle, requiring familiarity with the individual animal—typically provided by the owner or primary caregiver [56]. Human subjectivity plays a major role in how canine pain is perceived, interpreted, and managed. Both veterinarians and non-professionals bring their own beliefs, emotional responses, and biases to the assessment process, which can ultimately affect clinical decisions and animal welfare outcomes. Despite the availability of standardized scales, subjective perception remains a major challenge to consistent pain management in dogs. For instance, it is commonly assumed—without physiological evidence—that small-breed dogs are more sensitive to pain than larger breeds [57,58]. Moreover, individual canine traits such as extraversion may influence behavioral pain scores, while owners generally fail to predict these variations accurately [59]. Human attitudes toward dogs also interact bidirectionally with pain perception: anthropomorphic views may heighten empathy and even alter human pain tolerance, shaping how canine suffering is interpreted [60,61,62]. These subjective factors lead to inconsistent identification and management of pain in clinical practice. To mitigate such bias, incorporating measures that reflect the psychological disposition or treatment expectations of the owner may be valuable. In this regard, treatment expectation scales can serve as indirect indicators of owner mindset and help contextualize behavioral pain evaluations [8].

## 2. Objectives

The primary aim of this study protocol is to investigate the therapeutic potential of low-dose 1-cyclopropionyl-D-lysergic acid diethylamide (1cp-LSD), a legal LSD analog in certain countries, in managing chronic pain in dogs with osteoarthritis. A secondary aim is to evaluate treatment expectancy as an indirect indicator of potential placebo effects during the experimental protocol. By clearly defining these objectives, the proposal provides a structured framework for assessing both pharmacological efficacy and the psychological context of treatment, supporting subsequent experimental design and interpretation of results.

Although 1cp-LSD acts as a prodrug of LSD following metabolic cleavage [63], its pharmacokinetic and pharmacodynamic characteristics have not yet been characterized in humans or in any animal species. As a consequence, potential species-specific differences in absorption, metabolic pathways, receptor distribution, and binding affinity remain entirely unknown. This uncertainty underscores the exploratory nature of the present protocol and reinforces the need to generate foundational data in dogs—particularly in light of the growing interest in the therapeutic use of lysergamides in veterinary medicine [54,55].

## 3. Experimental Design

### 3.1. Study Design

The study will follow a randomized controlled trial design with a post-treatment follow-up phase. Dogs diagnosed with osteoarthritis will be randomly assigned to either the treatment group, receiving low-dose 1cp-LSD, or the control group, receiving placebo. The diagnosis of OA will follow the diagnostic algorithm recommended by the Canine Chronic Pain Expert Committee [64], which includes: (i) clinical observation, followed by (ii) orthopedic examination, (iii) complementary diagnostic tests (radiography and arthrocentesis), and (iv) advanced imaging procedures (ultrasonography, computed tomography, and/or magnetic resonance imaging). Randomization will be conducted using a computer-generated allocation sequence stratified by sex, body weight, and baseline pain level to ensure a balanced distribution between treatment arms. Allocation concealment will be ensured using sequentially numbered, opaque, sealed envelopes prepared by an independent researcher not involved in enrollment or assessment activities. Blinding will be implemented at multiple levels: clinical evaluators and investigators responsible for outcome assessments will be blinded to group allocation; caregivers administering the intervention will be blinded, with the active drug and placebo prepared to be visually indistinguishable; and data analysts will remain blinded to group identities until primary data analyses are completed. Treatment assignment will be revealed only at the end of the study. These procedures are designed to mitigate potential biases and preserve the integrity of the trial.

**Figure 2:**
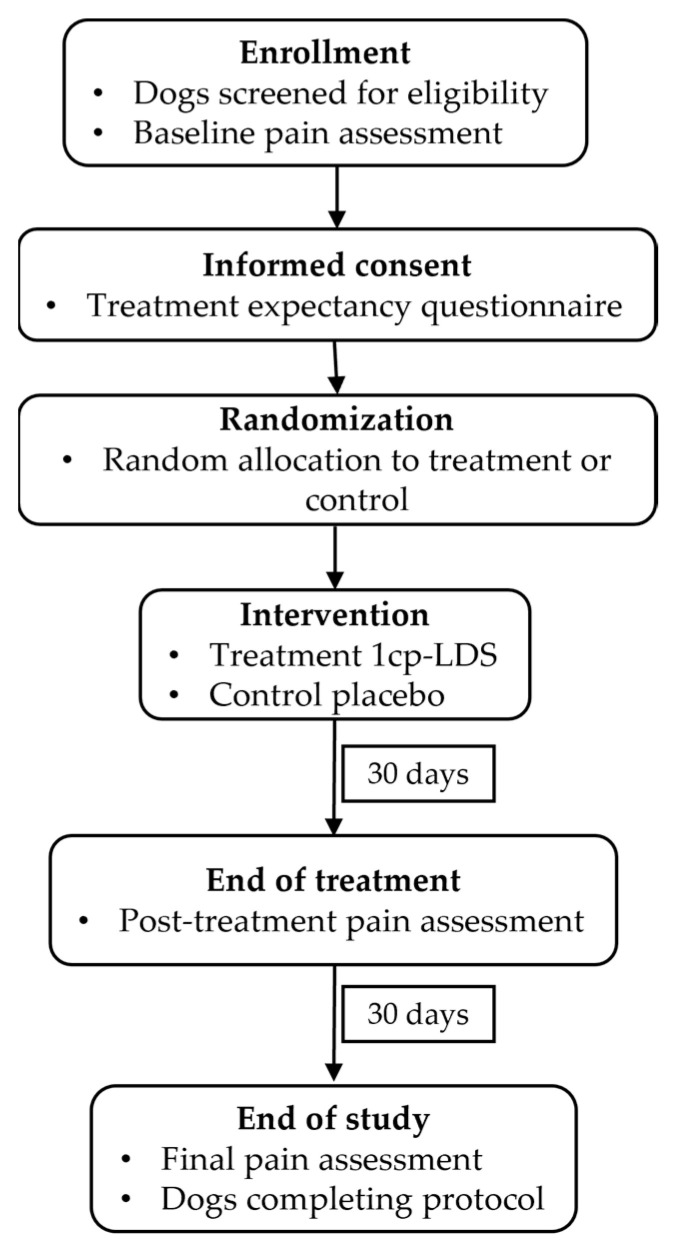
CONSORT-style flow diagram summarizing the experimental design. Dogs diagnosed with osteoarthritis will be randomly allocated to receive either low-dose 1cp-LSD or placebo. Evaluations will be performed at baseline (T0), end of treatment (T1), and post-treatment follow-up (T2). The design allows assessment of both immediate and sustained effects on pain and mobility outcomes.

The individuals will be assessed repeatedly at predefined time points—before, during, and after treatment—to monitor both short- and long-term effects. This design enables the evaluation of immediate and sustained therapeutic outcomes, including changes in pain-related behavior and mobility. A follow-up period will be included to determine the persistence of potential benefits after treatment withdrawal. Figure 2 shows an overview of the experimental timeline, including randomization, treatment phases, and assessment points.

To ensure animal welfare and protocol integrity, predefined monitoring steps will be applied throughout the study. All enrolled dogs will be clinically checked at regular intervals during the treatment phase, as well as during the follow-up period. Additional evaluations will be conducted whenever clinical signs worsen or if any adverse event is suspected. Dog owners will receive clear instructions on how to identify signs of discomfort or unexpected changes in their animals’ behavior or health status. They will be able to contact the attending veterinarian at any time during the study to report concerns, request guidance, or trigger an unscheduled examination if necessary. All such contacts and interventions will be documented and considered in the overall assessment of safety and tolerability.

### 3.2. Study Population and Proposed Sample Size

Dogs diagnosed with osteoarthritis will be enrolled in this pilot study and assigned to either the 1cp-LSD intervention or a placebo (saccharin) control group. Breed and sex will not serve as exclusion criteria. Eligible dogs must have a clinical diagnosis of osteoarthritis confirmed by a veterinarian, irrespective of the anatomical location of the affected joint(s). Severity and the duration since the initial diagnosis of osteoarthritis will be recorded but will not be a limiting factor for inclusion. Owners must provide informed consent to participate in the study.

To safeguard animal well-being, all dogs receiving standard analgesic treatment for osteoarthritis will maintain their prescribed medications throughout the study period. Although this approach may introduce variability, the withdrawal of established pain management would be ethically unacceptable in animals suffering from chronic pain. To minimize potential confounding, animals will be stratified based on their baseline analgesic regimen and allocated as homogeneously as possible between treatment groups. This strategy aims to ensure comparability across groups and to allow the evaluation of any additive or independent therapeutic effect of 1cp-LSD alongside ongoing standard care. Severity and time since diagnosis will be controlled by ensuring balanced distribution between treatment groups through stratified randomization.

For safety reasons, the following animals will be excluded: (i) dogs under 1 year of age, (ii) dogs with severe cardiac or hepatic disease, and/or (iii) dogs of breeds classified as potentially dangerous under Spanish Law 50/1999 (23 December). Due to the interaction between opioids and serotonin, dogs receiving opioid-based analgesia will be excluded. Dogs treated with amitriptyline or other drugs that interact with monoaminergic systems will likewise be excluded from the study.

Considering the absence of prior empirical data assessing 1cp-LSD administration in canine osteoarthritis, the determination of sample size proposed has been guided by methodological principles rather than formal power calculations. Accordingly, a randomized, parallel-group, placebo-controlled pilot design has been adopted, with 12 subjects allocated per arm (*n* = 24 in total). This sample size aligns with established recommendations for pilot studies, as it is sufficient to yield preliminary estimates of variability and mean changes in continuous outcome measures with acceptable precision, while preserving feasibility and ethical proportionality within the clinical veterinary context [65,66]. Such a design facilitates the refinement of outcome assessments, evaluation of procedural feasibility, and generation of variance estimates essential for informing formal sample size calculations in subsequent confirmatory efficacy trials.

### 3.3. Assessment Instruments

#### 3.3.1. Assessment of Chronic Pain in Dogs

Chronic pain will be evaluated using the Canine Brief Pain Inventory (CBPI), a validated owner-reported questionnaire specifically developed for assessing pain associated with osteoarthritis [67]. The instrument includes ten items rated on an 11-point Likert scale (0–10), addressing both pain severity and its interference with daily activities, along with an additional global item assessing the dog’s overall quality of life on a 5-point scale (Poor–Excellent). The CBPI has demonstrated high internal consistency and sensitivity to changes following therapeutic intervention, making it particularly suitable for monitoring treatment response in clinical trials involving chronic pain [68]. This information will complement the clinical assessment of all enrolled dogs, which will be performed by veterinary professionals in the context of routine clinical practice for osteoarthritis management.

#### 3.3.2. Assessment of Caregiver Treatment Expectations

Caregiver-related expectations regarding treatment outcomes will be assessed using the Treatment Expectancy Questionnaire (TEX-Q), a psychometrically validated instrument that explores the perceived credibility and anticipated efficacy of the proposed intervention [69,70]. The questionnaire consists of 15 core items and 6 supplementary questions rated on an 11-point Likert scale, providing a multidimensional measure of expectancy that may influence both caregiver perception and treatment adherence.

### 3.4. Treatment Protocol

Dogs assigned to the treatment group will receive an oral dose of 1cp-LSD. Because pharmacokinetic data for dogs are currently unavailable, dose selection will be guided by a human microdosing framework (20–40 µg per administration), as previously reported [55]. To adapt this range to canine subjects of different sizes, the human-equivalent dose will be calculated using an extrapolative approach based on body surface area (BSA). For each dog, BSA will be estimated using both body weight and body length, and normalized to a standard human of 80 kg and 180 cm. This method will provide a physiologically informed approximation of exposure and will avoid the limitations of fixed per-animal dosing. This dosing range has previously been evaluated in the canine species, showing no clinically relevant psychoactive manifestations or adverse effects [53,54,55]. 1cp-LSD will be sourced legally from a certified online vendor (AlphaChain B.V., Utrecht, The Netherlands). Each formulated unit contain 10 µg of 1cp-LSD L-tartrate. The supplier will provide a certificate of analysis documenting compound identity, concentration and purity as determined by quantitative nuclear magnetic resonance spectroscopy performed at manufacture. The compound will be administered once every three days for a total of ten administrations, resulting in a treatment period of 30 days. Each dose will be orally delivered by concealing the tablet within a small portion of palatable wet food to ensure voluntary ingestion and minimize handling-related stress. Following completion of the dosing phase, animals will enter a 30-day follow-up period, yielding a total study duration of 60 days (Figure 2). Dogs in the placebo group will be managed under identical conditions. Owners, who will be responsible for administering the medication, will remain blinded to their animal’s group assignment throughout the entire study.

Prior to treatment initiation, owners will complete all assessment instruments described in Section 3.3. The same instruments, excluding the Treatment Expectancy Questionnaire (TEX-Q), will be administered at the end of the treatment period and after the follow-up phase (Figure 2). This repeated-measures design will enable the evaluation of changes in pain perception over time while controlling for expectancy effects.

### 3.5. Contingency Plan and Rescue Analgesia

Adverse events will be systematically monitored and documented throughout the study duration. Clinical personnel will conduct continuous observation during and immediately after treatment administration, with follow-up assessments scheduled at predetermined intervals to detect any delayed reactions. All adverse events will be recorded using standardized case report forms, noting onset time, duration, severity, and suspected causality relative to the study intervention. In the event of any serious adverse event, appropriate clinical decisions will be made promptly, prioritizing animal welfare above all other considerations. This comprehensive monitoring protocol is intended to ensure participant safety and data integrity.

Rescue analgesia will be considered necessary when a clinically relevant deterioration relative to each dog’s baseline pain level is detected—whether through CBPI scores, orthopedic evaluation, or the attending clinician’s judgment—such that modification of the initial treatment conditions becomes ethically required. In case of pain exacerbation or insufficient analgesic control, dogs will receive rescue analgesia according to standard clinical practice [16]. A short-acting opioid (e.g., buprenorphine) will be used as first-line rescue medication, while NSAIDs will be maintained or adjusted as clinically indicated by the supervising veterinarian. However, if opioid administration is deemed necessary, the animal will be withdrawn from the study, as opioids interact with serotonergic pathways [71] and could confound the neuromodulatory effects under investigation. All instances of rescue analgesia, including the decision-making criteria and timing, will be documented for transparency and interpretation of results.

### 3.6. Data Collection and Statistical Analysis

All data will be collected prospectively throughout the study period. Pain and quality-of-life scores obtained from the CBPI will be recorded at baseline, at the end of the treatment phase, and following the 30-day follow-up period. Treatment expectancy data from the TEX-Q will be collected exclusively at baseline to prevent bias arising from repeated administration.

Descriptive statistics will be calculated for all variables, including means and standard deviations for continuous data. Normality will be assessed using the Shapiro–Wilk test. To evaluate treatment effects over time while accounting for individual variability, delta (Δ) values representing changes between consecutive time points will be computed. These Δ values will be used for subsequent analyses. Group comparisons will be performed using Student’s *t*-test. Non-parametric tests will be applied when normality assumptions are not met (e.g., Mann–Whitney U test), ensuring robustness of the statistical inferences. Correlations between continuous variables will be examined with Pearson’s r. Linear regression models will be applied to explore associations with changes in outcomes. Statistical significance will be set at *p* < 0.05 (two-tailed). Given the exploratory nature and limited sample size, effect sizes (e.g., Cohen’s d) will complement *p*-values to better interpret findings. Missing data and dropouts will be carefully recorded and reported. The primary analyses will be conducted on a per-protocol basis, excluding subjects who do not complete the study. Sensitivity analyses may be considered to evaluate the impact of missing data on the results. Data management and analyses will be conducted using PASW Statistics (version 19.0, SPSS Inc., Chicago, IL, USA). A similar analytical approach has been previously implemented in experimental studies investigating low-dose 1cp-LSD treatment for canine anxiety [55].

### 3.7. Ethical Considerations

Given the exploratory and pioneering nature of this proposed protocol, particular attention will be paid to ethical, methodological, and welfare aspects throughout its planning and design.

The study would involve privately owned companion dogs living in their home environments, under the continuous supervision of their caregivers. In accordance with current European and Spanish legislation, these animals would not be considered experimental subjects, as they would participate voluntarily in non-invasive procedures with informed owner consent. The study would require explicit approval from the relevant Institutional Animal Experimentation Ethics Committee, including authorization of the corresponding Owner Information Sheet and Informed Consent Form. Informed consent will be obtained in writing from all dog owners prior to inclusion, with a clear explanation of study procedures, potential risks, and benefits. Owners will be informed of their right to withdraw their animals from the study at any time without penalty. Criteria for early withdrawal will include any significant worsening of pain, adverse reactions, or other welfare concerns as assessed by the attending veterinarian. In such cases, appropriate clinical management will be prioritized, and data from withdrawn subjects will be handled according to the predefined analysis plan. Regular communication between owners and veterinarians will be maintained throughout the study to promptly identify any issues warranting withdrawal.

The sample size would be limited in view of the ethical implications of administering an unlicensed psychoactive compound in a veterinary context. Comparable studies previously published in the literature have also employed small cohorts, which is common and often necessary in preliminary pharmacological research [55,72,73]. This proposed design would therefore prioritize both scientific validity and the protection of animal welfare and owner trust.

To safeguard animal well-being, any dogs receiving standard analgesic treatment for osteoarthritis will maintain their prescribed medication throughout the study. While pharmacological interactions between 1cp-LSD and commonly used nonsteroidal anti-inflammatory drugs (NSAIDs) are not anticipated [74], the interactions of 1cp-LSD with other medications remain incompletely understood. However, dogs currently treated with drugs acting on monoaminergic systems, such as tramadol or amitriptyline [75], will be excluded from the study to minimize potential confounding effects and ensure the validity of the results.

The study has to be conducted in accordance with the ethical principles outlined in the European Directive 2010/63/EU on the protection of animals used for scientific purposes, and it has to adhered to the reporting standards recommended by the Animal Research: Reporting of In Vivo Experiments (ARRIVE) guidelines [76,77]. Prior approval from the relevant Institutional Animal Experimentation Ethics Committee (Institutional Review Board) will be required before any study procedures or data collection are initiated. Informed consent will be obtained prior to inclusion, with detailed explanations of study procedures, data collection methods, potential risks, and benefits.

## 4. Expected Outcomes and Limitations

It is anticipated that dogs receiving low-dose 1cp-LSD will experience a reduction in chronic pain, consistent with the proposed neuroplastic and serotonergic mechanisms attributed to psychedelic compounds in pain modulation [45,47]. Although a sustained effect of psychedelics beyond the treatment phase has been observed in the context of canine anxiety [54,55], the persistence of such effects in osteoarthritis—where interactions with inflammatory and pain mediators are involved—remains uncertain and, while desirable, lacks solid scientific evidence.

Scores obtained from the TEX-Q, reflecting caregivers’ treatment expectations, may influence their assessment of their pets’ pain and quality of life. The individual characteristics of each caregiver and the quality of their relationship with their pet may even influence the overall success of pharmacological treatments [55]. Moreover, given that perceptions of animal well-being are affected by multiple factors [78,79], these represent potential sources of bias that must be carefully considered and analyzed descriptively.

Several limitations should be considered. First, the optimal therapeutic dose in canines remains unknown, and the selected range (5–10 µg per administration) is based on safety considerations and previous studies [53,54,55]. Second, the treatment protocol—ten doses administered every three days over 30 days—may not capture the full potential effect of 1cp-LSD, as no dose–time studies exist in this species. Nevertheless, the schedule was informed by prior evidence in canine anxiety management [54,55]. Third, the study would include a carefully determined number of animals in accordance with ethical considerations and the principles of the 3Rs, aiming to minimize animal use while maintaining scientific validity [55,72,73]. No adverse effects are expected at the proposed low doses of 1cp-LSD [39,55]. Particular attention will be given to ensuring that treatment and control groups are as homogeneous and comparable as possible in terms of breed, baseline analgesic regimen, severity of osteoarthritis, and any other variables that could represent potential sources of bias within the selected cohort. Fourth, to safeguard animal well-being and to minimize the number of animals used in this exploratory phase, all enrolled dogs will maintain their prescribed standard analgesic treatment throughout the study. Concomitant medications will be carefully recorded to monitor potential confounding and adverse effects. Consequently, an active comparator with established efficacy is not included in this protocol. While this approach is ethically justified, it introduces a methodological limitation, as the absence of a direct active control precludes comparative efficacy assessment at this stage. The inclusion of an active comparator will be considered in future confirmatory trials once safety and preliminary efficacy have been demonstrated, thereby balancing ethical considerations with rigorous scientific evaluation. Finally, owner-reported outcome measures (CBPI, TEX-Q) inevitably involve a degree of subjectivity. Nonetheless, this limitation is explicitly acknowledged and is integral to the protocol’s secondary objective of assessing treatment expectancy and its potential influence on perceived analgesic outcomes. By quantifying this expectancy-related bias, the study aims to contextualize subjective reports and strengthen the interpretation of preliminary efficacy signals.

Potential regulatory and legal implications represent significant considerations in the clinical application of psychedelics within veterinary medicine. Psychedelic compounds are classified as controlled substances in numerous jurisdictions, imposing strict regulations on their manufacture, possession, and use. 1cp-LSD is considered a prodrug or legal analogue of LSD in many countries, though not universally. Such legal analogues often serve as practical alternatives in exploratory research, allowing preliminary investigation while navigating regulatory restrictions [55]. Consequently, clinical research involving these agents requires careful adherence to varying regulatory frameworks and ethical standards, which may limit subject availability, drug access, and study generalizability. Given the variable legal status of 1cp-LSD across countries, investigators should carefully assess and comply with local regulations prior to use. Recognition of these challenges is essential to contextualize the study outcomes and guide responsible future research in this emerging field.

## 5. Conclusions

Taken together, this protocol outlines a conceptual and methodological framework for investigating the potential analgesic effects of low-dose 1cp-LSD in dogs with osteoarthritis. Rather than presenting empirical findings, the proposed design aims to articulate testable hypotheses, refine dosing and assessment strategies, and clarify the role of caregiver-related factors that may influence outcome evaluation. By establishing a structured foundation, this protocol is intended to guide future empirical studies. Larger, controlled trials with extended follow-up will ultimately be required to confirm safety, efficacy, and the potential contribution of this compound to canine welfare.

## Data Availability

The original contributions presented in this study are included in the article. Further inquiries can be directed to the corresponding author.
